# Sexual Dysfunctions in Females with Parkinson’s Disease: A Cross-Sectional Study with a Psycho-Endocrinological Perspective

**DOI:** 10.3390/medicina59050845

**Published:** 2023-04-27

**Authors:** Rosaria De Luca, Mirjam Bonanno, Elisabetta Morini, Angela Marra, Francesca Antonia Arcadi, Angelo Quartarone, Rocco Salvatore Calabrò

**Affiliations:** IRCCS Centro Neurolesi “Bonino-Pulejo”, Via Palermo, SS 113, C. da Casazza, 98123 Messina, Italy; rosaria.deluca@irccsme.it (R.D.L.); elisabetta.morini@irccsme.it (E.M.); angela.marra@irccsme.it (A.M.); francesca.arcadi@irccsme.it (F.A.A.); angelo.quartarone@irccsme.it (A.Q.); roccos.calabro@irccsme.it (R.S.C.)

**Keywords:** sexual dysfunctions, female, Parkinson’s disease, psycho-endocrinological perspective, frequency of sexual dysfunction

## Abstract

*Background and Objectives*: Normal human sexual functioning is a complex integration of an intact neuroanatomic substrate, vascular supply, a balanced hormonal profile, and a predominance of excitatory over inhibitory psychological mechanisms. However, sexual functioning in Parkinson’s disease (PD) is often overlooked in clinical practice, especially in female patients. *Materials and Methods*: In this cross-sectional study, we have investigated the frequency of sexual dysfunction and the possible correlation with psycho-endocrinological factors in a sample of women with idiopathic PD. Patients were assessed using a semi-structured sexual interview, in addition to psychometric tools, including the Hamilton Rating Scale for Anxiety and for Depression and the Coping Orientation to the Problems Experiences—New Italian Version. Specific blood tests, including testosterone, follicle-stimulating hormone (FSH), luteinizing hormone (LH), estrogen E2, prolactin (PRL), and vitamin D3 were also evaluated. *Results*: Our results reported a statistical difference in sexual intercourse frequency before and after the onset of PD (*p* < 0.001). The percentage of women who complained about reduced sexual desire increased after diagnosis (52.7%) compared to the period before the onset of the illness (36.8%). The endocrinological profile in females with PD revealed statistically significant differences regarding testosterone (*p* < 0.0006), estradiol (*p* < 0.00), vitamin D3 (*p* < 0.006), and calcium (0.002). Depression (44% characterized by perceived feelings of anger and frustration during sexual intercourse) and anxiety symptoms (29.5% reported feelings of fear and anxiety for not satisfying the partner) with abnormal coping strategies (48.14% experienced feelings of anger and intolerance) were also found to be statistically significant. This study showed a high frequency of sexual dysfunction in female patients with PD, which correlated with sexual hormone abnormalities, mood/anxiety, and coping strategies alterations. This supports the idea that there is a need to better investigate the sexual function of female patients with PD to provide them with an adequate therapeutic approach and potentially improve quality of life.

## 1. Introduction

Parkinson’s disease (PD) is the second most common age-related neurodegenerative disorder, affecting about 3% of the population by the age of 65 and up to 5% of the people over 85 years [1,2]. PD causes a progressive neuronal dopaminergic loss in the substantia nigra compacta and the storage of alpha-synuclein-positive cytoplasmic inclusions termed Lewy bodies [3,4]. The role of biological sex is an important factor in the development of PD. Indeed, sex-based differences have been found for factors that influence the development and phenotypical expression as well as the life prognosis of patients with PD [5,6]. Epidemiological and clinical features highlight the clear presence of sex-related differences, per PD, which affects men twice more often than women [7,8,9], but women have a higher mortality rate and faster progression of the disease [10,11]. There are also sex-related differences in the response to treatments for motor and non-motor symptoms, and the disease risk factors burden is different between women and men [12,13]. Altogether, these differences in PD support the idea that disease development might involve men and women with the same pathological mechanisms but in different ways [14,15]. The most common non-motor symptoms associated with the disorder include autonomic dysfunctions (orthostatic hypotension, genito-urinary problems, and stypsis), cognitive abnormalities, psychiatric symptoms, such as anxiety, depression, and apathy, and sleep disorders, including insomnia and REM behavior disorder [16,17,18,19]. Martinez-Martin and colleagues carried out a complex study in a cohort of 951 PD patients to assess the prevalence and severity of non-motor symptoms according to biological sex. They stated that fatigue, depression, restless legs, constipation, pain, loss of taste or smell, weight change, and excessive sweating were more severe and frequent in women than men [20]. The relationship between the female gender and pain has been recently confirmed in a large clinical study; indeed, together with affective and autonomic symptoms, motor complications, and younger age, the female gender predicts overall pain severity [21,22]. Regarding PD psychological alterations, the female gender is more susceptible to developing severe, persistent, and episodic anxiety and depression than men [23,24,25].

Sexual dysfunction (SD) is a common but still neglected concern in patients with PD [26,27]. Decreased libido and difficulties reaching orgasms are the most common SD in women with PD [28,29,30]. In the general population, SD occurs with a prevalence of about 43% in women and 31% in men [31]. In female PD patients, SD is present in up to 87% [32], with a negative impact on partnership [33,34]. Moreover, SD, such as lack of desire, is often associated with endocrinological alterations in estradiol, testosterone, prolactin, and sex-hormone-binding protein (SHBG) levels [35,36,37,38]. Some studies reported that females affected by PD could have deficient lubrication, dyspareunia, and problems with orgasm, vaginal tightness, involuntary urination, anxiety, and inhibition compared to matched controls [39,40,41].

Sexual desire depends, among others, on neurotransmitters, steroid hormones, vasoactive agents, and molecules that act through specific receptors at the peripheral and cerebral levels [42,43]. The influence of endogenous sex hormones and the correlation with non-motor symptoms, including behavioral and sexual problems, has been, to date, poorly investigated in females with PD [44,45]. Sexual health is a fundamental issue for both neurological patients and their partners because it affects psychological wellbeing and quality of life. Then, assessing SD in females with PD is particularly important to guarantee a complete support and multidimensional therapeutic approach in these patients.

The purpose of this cross-sectional study is to describe the frequency of SDs in females affected by PD, taking into consideration the patient’s psycho-endocrinological profile.

## 2. Materials and Methods

### 2.1. Study Population and Setting

A total of 38 (15 pre-menopausal and 23 post-menopausal) female PD patients with a mean age of 56.47 ± 10.51, diagnosed according to UK Parkinson’s Disease Society Brain Bank clinical diagnostic criteria [46], were consecutively recruited from the Outpatient Movement Disorders Clinic of the IRCCS Centro Neurolesi “Bonino-Pulejo” (Messina, Italy) between January and October 2022.

Female gender, age between 35 and 80 years, a Hoehn–Yahr stage of 1–3, and being on stable dopaminergic therapy for at least 6 months before enrolment were the inclusion criteria. Patients were excluded if they had severe endocrine–metabolic pathologies, endometriosis, pudendal neuralgia, and/or took drugs (i.e., antidepressants and antipsychotics) potentially affecting sexual function.

The local Ethics Committee approved this study (IRCCSME-18/2021) on 15 October 2021. Written informed consent was obtained from all participants, and the principles outlined in the Declaration of Helsinki were followed.

### 2.2. Procedures

The assessment was conducted according to the “on” phase of PD patients, on the same day of the routine neurological consultation by a multiprofessional team composed of a neurologist/sexologist, an endocrinologist, and a psychiatric rehabilitation technician. This latter administered the psychometric evaluation, the endocrinologist prescribed specific hormonal and vitamin dosages to investigate those abnormalities potentially affecting wellbeing and sexual function [47], while the neurologist/sexologist carried out the neurological examination and sexual consultation. The frequency of sexual dysfunctions was investigated via the administration of a specific questionnaire. Social and demographic information, including age, ethnic background, residency (rural or urban), educational level, religion, and marital status, were also collected.

### 2.3. Outcome Measures

The sexual questionnaire (a semi-structured interview created by the research team in the native language of the patients, i.e., Italian, and translated into English only for publication purposes; see Supplementary Materials) is composed of twenty-eight questions focused on specific sexual topic about the woman’s sexual life, including sexual desire, arousal, lubrication, orgasm, satisfaction, frequency and quality of sexual intercourse, and intimacy experience [48]. In addition, it investigates some behavioral aspects, such as mood, anxiety during sexual intercourse, and specific opinions/beliefs on the influence of the disease on sexual life and the level of satisfaction. Information about patients’ sexuality (i.e., sexual desire, sexual intercourse frequency, etc.) was related to two different periods: before diagnosis and after diagnosis. Notably, Questions 13 and 14 were asked to the partners (when applicable) to better understand relationship problems. Answers were either dichotomic (yes or no) or as open. However, for specific questions, a 5-point Likert scale was used. In particular, the scale was applied to Item 5, investigating the quality of relationship as well as to Items 17–22 assessing the level of satisfaction about the relationship with the partner. Regarding Items 25 and 27 that evaluate SD (pre- and post-morbid sexual desire) in relation to the PD’s diagnosis, the patient must assign a score from 1 to 3 (1 = absent/reduced sexual desire; 2 = poor, but present; 3 = preserved). We decided to use this specific interview to collect more information about patients’ disease-related sexual life and their satisfaction degree regarding their relationships, which is not present in other validated tools. Although the semi-structured interview has not been validated, it has been used in other neurological conditions as a complementary and valuable tool [31,47,48]. To avoid incomplete responses and to ensure the comprehensibility of each question, psychologists read and explained the whole interview as clearly as possible [31,48].

Severity of depression was assessed using the Hamilton Rating Scale for Depression (HRS-D), which is designed to rate mood- and depression-related symptomatology by investigating 21 areas. The questionnaire is conceived for adults and rates the severity of depression by probing mood, feelings of guilt, suicide ideation, insomnia, agitation or retardation, anxiety, weight loss, and somatic symptoms. The patient’s score is calculated on the first 17 answers [49].

Anxiety symptoms were investigated using the Hamilton Rating Scale for Anxiety (HAM-A), a clinician-based questionnaire that can be administered as a self-scored survey. It consists of 14 core symptoms and accounts for both psychological and somatic symptoms, comprising anxious mood; tension (including startle response, fatigability, and restlessness); fears (including of the dark/strangers/crowds); insomnia; ‘intellectual’ (poor memory/difficulty concentrating); depressed mood (including anhedonia); somatic symptoms (including aches and pains, stiffness, and bruxism); sensory (including tinnitus and blurred vision); cardiovascular (including tachycardia and palpitations); respiratory (including chest tightness and choking); gastrointestinal (including irritable bowel syndrome-type symptoms); genitourinary (including urinary frequency and loss of libido); autonomic (including dry mouth and tension headache) and observed behavior at interview (including restless, fidgety, etc.). Each item is scored from 0 (not present) to 4 (severe); >17/56 is the cut-off to indicate mild anxiety; 25–30 is considered moderate–severe [50].

The Coping Orientation to the Problems Experiences—New Italian Version (COPE-NIV), an improvement of the previous Italian version of the COPE, was administered to investigate coping styles in different contexts. It included five large essentially independent dimensions: social support, avoidance strategies, positive attitude, problem solving, and turning to religion. Altogether, these five dimensions are reliable and stable in time without any essential differences in relation to the level of education, gender, and age. Avoidance strategies were found to be the condition largely associated with emotional distress, whereas positive attitude and problem solving were related to less distress and greater psychological wellbeing. Lastly, social support and turning to religion were not associated with psychological wellbeing. [51].

To complete the multi-specialist assessment phase, the levels of Follicle-Stimulating Hormone (FSH), Luteinizing Hormone (LH), Estradiol (E2), Testosterone (TS), Prolactin (PRL), and Vitamin D3 (Vit. D3), besides standard blood tests, were prescribed and evaluated by the endocrinologist.

### 2.4. Statistical Analysis

The descriptive analysis of the sample was reported on demographic (Table 1), clinical, psychometric, and endocrinological variables (Table 2). In particular, continuous variables were expressed as mean ± standard deviation, while categorical data were expressed through frequencies and percentages. Based on the results of Shapiro normality test and the reduced sample size, we performed a non-parametric analysis. In detail, the Wilcoxon sum rank test was used to compare continuous variables (including age, endocrinological data, and psychometric outcome measures between pre-menopausal and post-menopausal women), and the χ^2^ test with continuity correction was used to assess statistical differences in proportions. The semi-structured interview contained multiple-choice questions used to collect data about patients’ sexual life before–after the illness, which were codified as ordinal variables at two different times as follows: before the diagnosis of illness (T0) and after the diagnosis of illness (T1). Linear correlations were also computed using Spearman’s rho coefficient. Analyses were performed on open-source software R 4.1.3 (R Foundation for Statistical Computer, Vienna, Austria) for Windows. Statistical significance was set at *p* < 0.05.

## 3. Results

### 3.1. Demographic and Clinical Features

A total of 38 female patients with a diagnosis of PD and a mean age of 56.47 ± 10.51 were enrolled in this study. No statistical significance was reported for demographic (Table 1), clinical, and psychometric variables. We distinguished between pre-menopausal (39.50%) with a mean age of 47 ± 6.55 and post-menopausal (60.52%) females with a mean age of 62.6 ± 7.58 PD women. In detail, demographic data showed that most women (65.7%) were married, although two of them did not have children. Then, the quality of satisfaction perceived was lower when it was related to their partners/friends compared to the relationship with parents (both mother and father) and brothers/sisters (see Table 1). From a clinical point of view, we found that PD females presented the following staging of the Hoehn and Yahr scale: 44.7% were in Phase 1; 23.6% were in Phase 1.5; 13% were in Phase 2; 2.63% were in Phase 2.5; 3% were in Phase 3. Moreover, patients were on stable therapy (i.e., without motor fluctuations, on–off phenomena, and dyskinesia): 45% took dopaminergic drugs, 40 % L-Dopa, and 15% both. The endocrinological profile in females (including pre- and post-menopausal) with PD revealed statistical significance concerning testosterone (*p* < 0.0006), estradiol (*p* < 0.00), vitamin D3 (*p* < 0.006), and calcium (0.002). For more detailed information about demographic, clinical, and other variables, see Table 1 and Table 2.

### 3.2. Sexual Dysfunction Characteristics

We found statistical differences in sexual intercourse frequency before and after the onset of PD (*p* < 0.001), while sexual desire does not reach statistical significance (*p* = 0.15). However, the percentage of women who complained about reduced sexual desire increased after diagnosis (52.7%), specifically in 73.3% of pre-menopausal women and 52.17% of post-menopausal women, rather than before the onset (36.8%) of the illness (see Table 3), with 40% in pre-menopausal women and 39% in post-menopausal females.

Nearly half of the PD sample (44%) perceived feelings of anger and frustration during sexual intercourse, and 29.5% of them reported also feelings of fear and anxiety about not satisfying their partner. From our interview, it emerged that 42% of the sample considered themselves less attractive to their partners, and 68.7% of these patients reported reduced sexual desire after the onset of PD. In addition, 55.6% of females perceived changes in their relationship with their partners after the diagnosis of PD. Specifically, 19.04% of them manifested frustration and intolerance for partners; 14.28% of patients felt feelings of embarrassment for their illness; 35.3% showed social isolation, distrust, and difficulty in relating with others; 6.8% of patients reported feeling anger; 10.3% felt more susceptibility; only 14.28% showed more humility and positive feelings. Moreover, patients referred to changes in their sexual intercourse linked to the illness due to motor alteration (10.52%), aging (15.78%), fear of betrayal (26.31%), and reduced intimacy and sexual desire (48.38%).

We also interviewed patients’ partners (using two dedicated questions of the same interview) to understand how they were experiencing the behavioral changes of the patients. Interestingly, we found that 71.05% of them reported feeling changes in females’ behavior: 48.14% stated that female partners experienced feelings of anger and intolerance (reduced coping strategies) and were more inclined to argue; 22.2% stated that their female partners had avoidant and closed attitudes; 18.51% of females showed depressive symptoms; only 11.15% of females were seen as more compliant and sensitive, according to partners’ opinion.

Eventually, we found a linear weak and negative correlation between HRS-A and LH values (rho = −0.30) and a moderate linear positive correlation between avoidance strategies of COPE and HRS-D (rho = 0.38).

## 4. Discussion

As far as we know, this is one of the few studies investigating sexuality in female PD patients and the first one focusing on psychological and endocrinological alterations to promote a multimodal approach and to better deal with these disabling problems.

SD is one of the most common, yet under-estimated, non-motor symptoms of PD, and it is often complicated by physical disability, affecting the quality of life of PD patients and their partners [52]. According to the current literature [53], our results showed that SD is highly relevant in female PD patients, especially in pre-menopausal women (<45 years and Hoehn–Yahr stage ≤ 2) [54]. However, SD was also found in 75.8% of older women, with a mean age of 65.1 and a Hoehn–Yahr stage of 2.6 (>10 years of disease) by other authors [55]. Additionally, epidemiological studies have pointed out that loss of libido and decreased sexual desire in PD women have a prevalence of 46.9 to 84% [56]. Another aspect that should be considered is the frequency of difficulties in arousal. In fact, some authors stated that difficulty in arousal or reaching orgasms was present in 87.5% of PD females with a mean age of 61.6 years and 8 years of disease duration. This was in contrast with younger women, who seem to have unaltered arousal and orgasm ability compared to controls [57]. Moreover, it has been found by Buhmann C. et al. [58] that women with PD reported more SD and orgasm-related problems during sexual intercourse, suggesting that partnership problems are more relevant for orgasm dysfunction than physical disorders. According to these findings, we investigated the degree of satisfaction with patients’ partners, which was very low compared to the relationship with relatives and friends. The data further suggest that the disease-related SD are not only due to neurobiological changes but also to changes in relationships, given that the diagnosis of a chronic illness affects mood as well as the patient’s perspective of his/her life. The mood is strictly related to sexuality in a bidirectional way, and the way one copes with the disease could be fundamental in determining SD [59]. In this vein, we have assessed coping strategies and found that avoidance is potentially correlated with SD. These findings, however, deserve further investigation.

Furthermore, we found that reduced libido was the most prevalent SD, in more than 50% of the sample, whereas in other studies SD prevalence was about 30%. Nevertheless, a recent meta-analysis [60] did not find a higher prevalence of SD in the female PD population compared to healthy controls. This discrepancy could be due to the different assessment tools used, including our specific semi-structured interview built to investigate SD in this specific patient population.

To better understand the etiology of PD-related SD, Varanda S. et al. [61] excluded from their study the psychological causes, although depression and anxiety are much more significant than other variables, as they are known to affect sexuality in the general population as well as in other neurodegenerative disorders [47]. Our multidisciplinary assessment approach (i.e., neurosexological, endocrinological, and psychological) helped us to better demonstrate the potential causes of SD [62]. Indeed, we found that the latter was primarily associated with abnormalities in sex hormone levels. Estrogen loss has been postulated to be associated with female SD in the elderly population since there were significant declines in sexual function by the postmenopausal phase [56]. Okun et al. have shown that a low testosterone level is a recognized cause of depression, fatigue, decreased libido, and decreased work performance [63], as also demonstrated in our study. Then, sex hormones such as estrogen and testosterone may play a crucial role in affecting sexual functioning in females with PD, although research in this area is still scarce [64]. Moreover, we found that in our female sample, there were low levels of Vitamin D3, although we are not able to state if and to which extent the data could be related to SD or associated symptoms. Vitamin D is important for brain development and brain activity, and it has been associated with many neurological diseases, including PD [65]. However, the results of studies investigating the association with non-motor symptoms are conflicting [66,67]. In persons with PD, depression and cognitive impairment seem to be influenced by vitamin D_3_, as reported by Peterson et al. 2013 [68]. Better scores in neuropsychiatric testing, especially verbal fluency and verbal memory, are associated with higher serum levels [69], and vitamin D3 concentration was correlated with depression and anxiety scores [70]. Moreover, SD itself is commonly associated with depression and relationship dissatisfaction, which contributes substantially to sexual unhappiness and reduced quality of life [71,72], as confirmed by our data. Then, an interplay between hormone levels, vitamin D3, and mood alterations should be considered when dealing with SD in female patients with PD. Moreover, coping strategies should also be evaluated in these patients, given that avoidance strategies may further affect sexuality and relationships, as in our sample.

According to the World Health Organization (WHO) (2006) [73], sexual health is a fundamental aspect of life, regardless of the presence of SD, and everyone has the right to conduct their relationships and have personal control over sexual and reproductive behavior with adequate information and treatment. Interestingly, some authors [74] investigated reproductive behavior and pregnancy in PD females. In this context, a pregnancy condition is not a frequent occurrence (since, usually, the disease appears at an older age); however, female patients should be informed of the possible worsening of their non-motor symptoms during pregnancy related to the disease progression of physiological changes and effects of estrogen. Nonetheless, it seems that there is not an increased risk of birth complications or fetal abnormalities in pregnant women with PD, although the assumptions of anti-parkinsonian drugs could complicate childbirth [75].

To sum up, our findings support the idea that sexual disorders in females with PD should be systematically screened and monitored, focusing also on psychological and endocrinological aspects, to reduce the incidence of these non-motor symptoms and for the better management of female patients with PD [76]. In line with some authors [77,78], we believe that early recognition with an adequate and complete treatment of non-motor symptoms of PD, including SD, is essential, owing to multifactorial and multidimensional repercussions on females with PD’s quality of life.

Our study has some limitations to knowledge. The sample is relatively small to extend the results to the general female with PD population, and a control group is missing to confer greater methodological rigor. However, data from the literature show a prevalence of loss of libido and decreased sexual desire even in young women with an early onset of PD, suggesting the important role of psychological factors that are more relevant at the onset of SD rather than in men. Nonetheless, in this cross-sectional study, we have investigated the SD-related psycho-endocrinological factors, including mood, anxiety, and coping strategies, beyond the sexual hormone level dosage. This novel approach was aimed to promote the implementation of a more systematic and complete management of these complex non-motor symptoms frequently affecting females with PD.

From a future perspective, it could be interesting if future studies investigate this important issue focusing on the correlations among age, post-menopausal status, stage of PD, motor scores using the UPDRS, and psychological alterations (i.e., depression and anxiety). In fact, we stated that depressive mood can affect female coping strategies, possibly avoiding sexual intercourse. This is why, future and larger studies should perform a multiple regression model to understand the main demographical and clinical features involved in the onset of SD in PD females. Additionally, future research could investigate the presence of SD by examining a matched sample of people of the same age and gender who do not have sexual dysfunction, which could be further helpful in exploring the factors associated with/predicting these disabling symptoms in these patients.

## 5. Conclusions

Sexual disorders are the most neglected non-motor symptoms in PD, especially in female patients. Indeed, with this study, we have shown that SD, including loss of libido, is a frequent concern in female subjects with PD, in contrast with the current literature. Given that SD could be associated with mood, behavior, and endocrinological disorders, as well as maladaptive coping strategies, we believe that there is a need for a multidisciplinary approach for a better management of these still overlooked non-motor symptoms. Further larger multicenter studies should be fostered to confirm such a high prevalence of SD in female PD patients and to better establish the role of the different factors in determining these disabling symptoms.

## Figures and Tables

**Table 1:** Demographic description of the sample.

Characteristics	All Female Patients	Pre-Menopausal Females	Post-Menopausal Females	*p*-Value
Age (Years)	56.47 ± 10.51	47 ± 6.55	62.6 ± 7.58	0.99
Education (In Years)				0.76
Elementary School	2 (5.27)	0 (0.00)	2 (8.69)	
Middle School	13 (34.21)	7 (46.6)	6 (26.08)	
High School	23 (60.52)	8 (53.3)	15 (65.2)	
Marital Status				0.37
Single	3 (7.89)	3 (20.00)	0 (0.00)	
Married	25 (65.7)	7 (46.6)	18 (78.2)	
Divorced	8 (21.05)	5 (33.3)	3 (13.04)	
Widowed	2 (5.27)	0 (0.00)	2 (8.69)	
Children				0.23
Yes	33 (86.84)	10 (66.6)	23 (100.00)	
None	5 * (13.15)	5 (44.4)	0 (0.00)	
	* two of five patients were married.			
Clinical Data				
Years of Illness				0.28
< 1 year	9 (23.6)	4 (26.6)	5 (21.73)	
1–5 years	13 (34.2)	3 (20.00)	11 (47.82)	
</= 10 years	7 (18.4)	4 (26.6)	3 (13.04)	
>10 years	9 (23.6)	5 (33.3)	4 (17.39)	
Hoehn and Yahr Stadiation	**1.53 ± 0.70**	1.4 ± 0.72	1.52 ± 0.77	0.80
Autonomic Symptoms				
				0.66
Hypotension	4 (10.5)	2 (13.33)	2 (8.69)	
Constipation	6 (15.7)	3 (20.00)	3 (13.04)	
Urinary Incontinence	2 (5.26)	1 (6.6)	1 (4.34)	
None	26 (68.4)	10 (66.6)	16 (69.56)	
Degree of Relationship Satisfaction (rated from 1 to 5) with:				
The Partner	3.8 ± 1.39	3.76 ± 1.48	3.9 ± 1.37	0.23
The Father	4.16 ± 1.20	4.2 ± 1.20	4.1 ± 1.23	0.38
The Mother	4.11 ± 1.40	4.3 ± 1.39	3.9 ± 1.43	0.42
Brothers/Sisters	3.9 ± 1.34	3.3 ± 1.71	4.4 ± 0.69	0.20
Friends	3.5 ± 1.02	3.6 ± 0.81	3.4 ± 1.16	0.45
Degree of Quality-of-Life Satisfaction	2.83 ± 1.20	2.5 ± 1.06	3.04 ± 1.28	0.50

Continuous variables were expressed as mean ± standard deviation, whereas categorical variables were as frequencies and percentages.

**Table 2:** Clinical, psychometric, and endocrinological characteristics of PD female sample.

Psychometric Evaluation	All Female Patients with a Diagnosis of PD	Pre-Menopausal Females	Post-Menopausal Females	*p*-Value
COPE subitems				
Social support	25.02 ± 5.25	**23.3 ± 5.39**	**26.13 ± 4.9**	0.99
Avoidance strategies	25.55 ± 5.10	**25.9 ± 4.49**	**25.3 ± 5.5**	0.99
Positive attitude	27.57 ± 6.54	**24.9 ± 7.43**	**29.3 ± 5.3**	0.99
Problem solving	28.57 ± 7.61	**29 ± 9.6**	**28.3 ± 6.21**	0.94
Turning to religion	23.81 ± 6.27	**22 ± 3.42**	**25 ± 2.9**	0.97
HRS-D	12.78 ± 6.72	**17 ± 7.38**	**12.95 ± 6.42**	0.93
HRS-A	10.23 ± 6.27	**11.2 ± 7.55**	**9.6 ± 5.38**	0.97
Endocrinological profile				
FSH	60.29 ± 25.2	**60.36 ± 29.18**	**63.45 ± 25.5**	0.92
LH	37.94 ± 15.01	**39.8 ± 16.69**	**36 ± 12.6**	0.87
PRL	17.66 ± 6.6	**18.26 ± 4.60**	**17.16 ± 8.4**	0.86
TS	20.21 ± 9.9	**19.8 ± 8.37**	**20.4 ± 11.14**	**0.0006**
E2	40.13 ± 30.5	**49.1 ± 14.1**	**22.3 ± 6.6**	**0.00**
Vit. D3	16.8 ± 6.10	**19.71 ± 6.29**	**15 ± 5.49**	**0.006**
Ca	9 ± 0.37	**8.72 ± 0.41**	**8.84 ± 0.35**	**0.002**

Legend: HRS-D (Hamilton Rating Scale—Depression), HRS-A (Hamilton Rating Scale—Anxiety), FSH (Follicle-Stimulating Hormone, 9–100 U/L), LH (Luteinizing Hormone, 15–62 mlU/mL), PRL (Prolactin, 4.7 a 23.3 ng/mL), TS (Testosterone, 6–25 ng/dL), E2 (Estradiol, <20 pg/mL), Vit.D3 (Vitamin D3, ng/mL >30), Ca (Calcium, 8.5–10.2 mg/dL).

**Table 3:** Description of prevalent Sexual Dysfunctions (SDs) in all females with PD.

Sexual Dysfunctions’ Features				*p*-Value
Sexual desire	Before illness		After illness	0.15
	No alteration 23 (60.5)		No alteration 15 (39.4)	
	Reduced 14 (36.8)		Reduced 20 (52.7)	
	Difficulty in reaching orgasm 1 (2.6)		Difficulty in reaching orgasm 3 (7.8)	
Sexual intercourse frequency	Before illness		After illness	0.001
	Less than once a month 5 (13.15)		Less than once a month 21 (55.2)	
	Monthly 15 (39.4)		Monthly 9 (23.6)	
	Bi-weekly 7 (18.5)		Bi-weekly 1 (2.63)	
	Weekly 10 (26.3)		Weekly 5 (13.2)	
	None 1 (2.6)		None 2 (5.2)	
Changes in relationship after diagnosis	Yes		No	0.75
	21 (55.6)		17 (44.7)	
Spontaneous or fantasy/thought-induced feelings of excitement	Yes		No	NA
	0 (0.00)		38 (100.00)	
Practice masturbation	Yes		No	NA
	4 (10.5)		34 (89.5)	
Feeling perceptions during sexual intercourse	Calmness	Indifference	Anger/frustration	0.67
	16(42.00)	6(15.00)	17(44.00)	

## Data Availability

Data will be available upon request to the corresponding author.

## Supplementary Materials

The following supporting information can be downloaded at: https://www.mdpi.com/article/10.3390/medicina59050845/s1.

## Abbreviations

Ca	Calcium
COPE-NIV	Coping Orientation to the Problems Experiences—New Italian Version
E2	Estradiol
FSH	Follicle-Stimulating Hormone
HRS-A	Hamilton Rating Scale—Anxiety
HRS-D	Hamilton Rating Scale—Depression
LH	Luteinizing Hormone
PD	Parkinson’s Disease
PRL	Prolactin
SD	Sexual Dysfunction
TS	Testosterone
Vit.D3	Vitamin D3
