# Supplementary material for: Sexual Dysfunctions in Females with Parkinson’s Disease: A Cross-Sectional Study with a Psycho-Endocrinological Perspective
Source: Medicina (Kaunas). 2023 Apr 27;59(5):845. doi: 10.3390/medicina59050845 (PMC10220810; doi:10.3390/medicina59050845)
Supplement: Supplementary file 1 [file medicina-59-00845-s001.zip › medicina-2326720-supplementary.pdf]

CENTRO NEUROLESI
BONINO PULEJO
IRCCS MESSINA
Istituto di Ricovero e Cura a Carattere Scientifico
Certificato n. 10271

PSYCHO-SEXOLOGICAL QUESTIONNAIRE

28-ITEMS SEMI-STRUCTURED INTERVIEW TO INVESTIGATE THE FREQUENCY OF SEXUAL DYSFUNCTIONS IN WOMEN WITH DIAGNOSIS OF PARKINSON'S DISEASE

Created In Italian by the multi-specialist team IRCCS Centro Neurolesi "Bonino Pulejo" of Messina , and Translated in English only for Publication purpose

SOCIO-DEMOGRAPHIC DATA

1. AGE ______ 2. Degree - EDUCATION (in years)________________________________

3. MARITAL STATUS ________________ 4. CHILDREN'S NUMBER ____________________________

FOR THE PATIENT (individual interview with the specialist)

5. For each of the following statements, indicate your corresponding degree of satisfaction, from 1 (minimum/very poor) to 5 (maximum/excellent).

Degree of satisfaction (from 1 to 5) - couple relationship: _____

Degree of satisfaction (from 1 to 5) - relationship with father: _____

Satisfaction degree (from 1 to 5) - relationship with mother: _____

Degree of satisfaction (from 1 to 5) - relationship with brothers/sisters: ______

Degree of satisfaction (from 1 to 5) - friendships: _____

Degree of satisfaction (from 1 to 5) - Quality of life (QoL): ______

6. Tick with an X autonomic symptoms presenting. Otherwise, nothing.

• constipation or diarrhoea.

• abdominal swelling and belching.

• low blood pressure (hypotension)

• dizziness and fainting when standing up.

• rapid heartbeat (tachycardia)

7. Please indicate how long you have been diagnosed with Parkinson's: ________________________________

8. Are you taking any medications for your neurological disease_____________________________________

9. Takes other drugs: ____

10. Has your relationship with others changed after illness? ____________________________________

11. If so, how? Specify whether for the better or for the worse? ______________________________________

12. If so, how much? (From 1 to 5) ____

FOR THE PARTNER/CAREGIVER (individual interview with the specialist)

13. Has changed patient's behaviour (communicative-relational) after the diagnosis?

If so, how? ____

14. The disease affects the sexual life?

If so, how? ____

FOR THE PATIENT (individual interview with the specialist)

15. You Practiced masturbation?

Yes or No

16. Do You have a stable emotional relationship?

Yes or No

____

17. If yes, to what extent do you feel satisfied with your relationship? (from 1 to 5) ____________________

18. Do you currently perceive yourself as attractive? (from 1 to 5)_____________

19. Do you think your partner considers you sexually attractive? (from 1 a 5) _____________________

20. Do you have spontaneous or fantasy/thought-induced feelings of arousal in the morning and/or throughout the day? Indicate the answer.

Yes or Not

21. How often before the illness did you have complete sexual intercourse? (1-5)______________________

22. And, after diagnosis, how often did you have sexual intercourse? (1-5)__________________________

23. How did you feel when thinking about future/ facing love affairs? ________________________________

24. Before the onset/diagnosis of the disease, did you have one or more sexual problems?

If yes, specify whether episodic or permanent: ____

25. PRE-DISEASE Sexual Desire IN NUMBER (1-3)_____________________________________

26. After the onset / diagnosis of the disease, did you have one or more of the sexual problems? ____

27. POST-DISEASE Sexual Desire IN NUMBER (1-3) _________

28. Evaluate and specify whether sexual symptoms did not arise after taking drugs to treat the neurological disease and/or drugs to treat associated symptoms:

Yes

Not

It is not clear
